# A Single 60.000 IU Dose of Erythropoietin Does Not Improve Short-Term Aerobic Exercise Performance in Healthy Subjects: A Randomized, Double-Blind, Placebo-Controlled Crossover Trial

**DOI:** 10.3389/fphys.2020.537389

**Published:** 2020-09-29

**Authors:** Thomas Haider, Victor Diaz, Jamie Albert, Maria Alvarez-Sanchez, Markus Thiersch, Marco Maggiorini, Matthias P. Hilty, Christina M. Spengler, Max Gassmann

**Affiliations:** ^1^Institute for Veterinary Physiology, Vetsuisse Faculty, University of Zurich, Zurich, Switzerland; ^2^Zurich Center for Integrative Human Physiology (ZIHP), Zurich, Switzerland; ^3^Department of Cardiology, University Hospital Zürich, Zurich, Switzerland; ^4^Institute of Human Movement Science and Sport, ETH Zürich, Zurich, Switzerland; ^5^Institute of Intensive Care Medicine, University Hospital of Zürich, Zurich, Switzerland

**Keywords:** EPO, high dose, oxygen transport, erythropoiesis, doping

## Abstract

Erythropoietin (EPO) boosts exercise performance through increase in oxygen transport capacity following regular administration of EPO but preclinical study results suggest that single high dose of EPO also may improve exercise capacity. Twenty-nine healthy subjects (14 males/15 females; age: 25 ± 3 years) were included in a randomized, double-blind, placebo-controlled crossover study to assess peak work load and cardiopulmonary variables during submaximal and maximal cycling tests following a single dose of 60.000 IU of recombinant erythropoietin (EPO) or placebo (PLA). Submaximal exercise at 40%/60% of peak work load revealed no main effect of EPO on oxygen uptake (27.9 ± 8.7 ml min^–1^⋅kg^–1^/ 37.1 ± 13.2 ml min^–1^⋅kg^–1^) versus PLA (25.2 ± 3.7 ml min^–1^⋅kg^–1^/ 33.1 ± 5.3 ml min^–1^⋅kg^–1^) condition (*p* = 0.447/*p* = 0.756). During maximal exercise peak work load (PLA: 3.5 ± 0.6 W⋅kg^–1^ vs. EPO: 3.5 ± 0.6 W kg^–1^, *p* = 0.892) and peak oxygen uptake (PLA: 45.1 ± 10.4 ml⋅min^–1^ kg^–1^ vs. EPO: 46.1 ± 14.2 ml⋅min^–1^ kg^–1^, *p* = 0.344) reached comparable values in the two treatment conditions. Other cardiopulmonary variables (ventilation, cardiac output, heart rate) also reached similar levels in the two treatment conditions. An interaction effect was found between treatment condition and sex resulting in higher peak oxygen consumption (*p* = 0.048) and ventilation (*p* = 0.044) in EPO-treated males. In conclusion, in a carefully conducted study using placebo-controlled design the present data failed to support the hypothesis that a single high dose of EPO has a measurable impact on work capacity in healthy subjects.

## Introduction

Erythropoietin (EPO), the master hormone regulating red blood cell (RBC) formation is commonly used in the clinics to treat anemia in patients with chronic kidney disease (Cernaro et al., 2019), heart failure (Anand and Gupta, 2018), and cancer (Cornes et al., 2007; Gilreath et al., 2014). In sports, EPO is well known for its potential to increase aerobic exercise capacity, which leads to its frequent misuse as performance enhancing drug in endurance-related sport disciplines (Momaya et al., 2015; Salamin et al., 2018). The “boosting” effect of EPO on exercise performance is believed to be mediated via the stimulation of erythropoiesis, which results in an increase in RBCs, total hemoglobin mass and subsequently oxygen transport capacity when administered regularly for several weeks (Lundby et al., 2008). However, in recent years other potentially ergogenic mechanisms, namely non-erythroid effects of EPO came into research focus and these effects were discussed to eventually affect exercise performance (Sgro et al., 2018). In this context, a recent preclinical study reported that both chronic cerebral EPO overexpression and a single high dose of systemic EPO treatment leading to elevated cerebral EPO levels were able to improve maximal and submaximal exercise performance in mice without alterations in RBC blood parameters such as blood volume, total hemoglobin mass and hematocrit (Schuler et al., 2012). It was concluded that EPO might exert its ergogenic effect potentially by central, none specified mechanisms within the brain. Indeed, EPO and its receptor were shown to be expressed in various non-erythroid tissues including the brain (Marti et al., 1997). Furthermore, it was shown that circulating EPO is able to cross the blood brain barrier and to elevate brain EPO levels as measured in the cerebrospinal fluid when administered as systemic single high-doses, ranging from 667 to 1500 IU⋅kg^–1^ (Xenocostas et al., 2005). Of importance, short-term high-dose EPO treatment was also reported to be safe and generally well tolerated with potential neuroprotective properties and anti-depressant-like effects in humans (Miskowiak et al., 2012). In this context, Miskowiak et al. (2008) could show that a single high EPO dose (40.000 IU) was able to increase cognitive function and emotional processing and furthermore improve mood ratings for three consecutive days in healthy volunteers. On the other hand, psychological factors such as mood state and motivation are known to modulate human exercise performance at a central- brain level (Lane and Terry, 1999, 2000). Thus, mood changes triggered by increased cerebral EPO concentrations may also have the potential to affect exercise performance. However, these findings were challenged by a recent randomized, double-blind, placebo-controlled crossover trial reporting that short-term high-dose EPO treatment (30.000 IU⋅day^–1^ for 3 consecutive days) – in contrast to the “classical” chronic EPO treatment regimen – had no ergogenic effect on exercise performance in healthy men (Rasmussen et al., 2010).

In the present study, we addressed this controversial aspect by performing a human trial with a comparable study design to test the hypothesis that a single – but higher – dose of EPO (60.000 IU) is able to improve mood, peak exercise capacity and endurance exercise performance in healthy males and females.

## Materials and Methods

### Ethical Approval

The study was approved by the Cantonal Ethical Commission of Zurich (KEK-ZH-NR: 2011-0170) and conducted in accordance with the declaration of Helsinki. All study participants gave their informed oral and written consent prior to the start of the experiments.

### Study Design and Study Subjects

The current investigation (exercise performance part) was part of the EPOPERF-project, a phase I/II randomized, double-blind, placebo-controlled, crossover single center study investigating the effects of a single high dose of 60.000 IU of EPO on cognitive function, respiratory control and exercise performance in healthy individuals (NCT01889056). During the first visit (screening visit) at the study site, a physician performed a short anamnestic interview with the study subjects, followed by a physical examination and a first venous blood sample was taken to assure inclusion criteria were met. Inclusion criteria were: Healthy males and females (age range of 18–35 years), non- smokers (≥1 year) with a normal body mass index of 18.5–24.9 kg m^–2^ and training state (no elite competitive athletes), able to perform cycling exercise. Main exclusion criteria were: Abnormal serum ferritin levels according to sex- and age-dependent laboratory reference values (normal reference values for age 18–60 years, males: 30–400 μg⋅l^–1^, females: 13–150 μg⋅l^–1^), a high hematocrit (>55%), pre-existing genetic homeostatic disorders (factor V Leiden mutation, prothrombin mutation), history of venous thromboembolic events, prolonged exposure (≥5 days) to high altitude (≥2,500 m) within the last 6 months prior to the study, non-compliant behavior, pregnant or breast feeding. On this first visit, subjects also performed the exercise tests to familiarize themselves to the testing regimen.

### EPO vs. Placebo Treatment and Health Monitoring

The study participants were randomly assigned, according to a 2 × 2 crossover design, to receive a short (15 min) intravenous infusion of either 60.000 IU of recombinant human (rh) EPO (Epoietin beta, Recormon, Roche Pharma AG, Switzerland) diluted in 250 ml 0.9% saline solution as EPO treatment condition (EPO), or an infusion of 250 ml 0.9% saline solution only as placebo treatment condition (PLA), 24 h prior to the start of testing. After a washout period of ≥ 4 weeks, subjects received the alternate treatment and, on the following day, all tests were repeated in the same order as performed on the first test day. The EPO-treatment dose chosen in the present study was based on previous studies, which reported that comparable doses of EPO were well-tolerated and safe to administer in healthy subjects while leading to increased cerebral EPO levels as previously shown by elevated EPO levels measured in the cerebrospinal fluid (Miskowiak et al., 2008; Rasmussen et al., 2010). Nevertheless, the subjects were health-monitored by regular measurement of blood pressure, heart rate, arterial oxygen saturation and subjective rating of well-being during the infusion period and for further 2 h post infusion. On the testing day (24 h after EPO- or placebo infusion), in the morning, subjects were asked to rate their sleep quality, mood and motivation. Then, cognitive tests and respiratory assessments were performed. In the afternoon, maximal exercise performance and endurance exercise performance were measured.

### Measurements

#### Subjective Rating of Sleep Quality, Mood, and Motivation

Subjective ratings (ranging from lowest = 0% to highest = 100%) of sleep quality, mood state and motivation level were assessed by use of visual analog scales (VAS). Additionally, the self-reported mood state was assessed by use of the positive (PAS) and negative (NAS) association scale (PANAS) (Watson et al., 1988), the scales previously used to assess the mood state after EPO treatment (Miskowiak et al., 2008).

#### Exercise Performance

**FIGURE 1:**
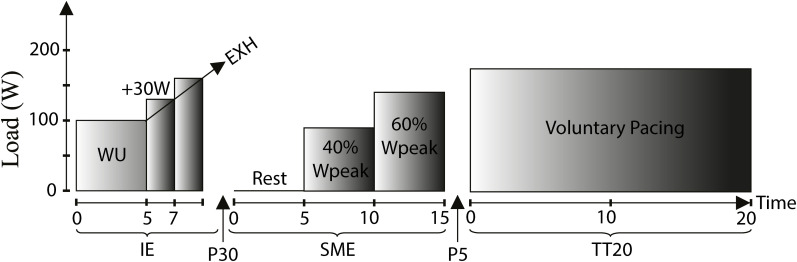
Exercise performance test protocol: Incremental exercise (IE), Submaximal exercise (SME), 20 min time trial (TT_20_), WU: Warm-up (males: 100 W/females: 70 W), Rest: Resting condition (baseline), 40%W_peak_: 40% of peak work load achieved during IE, 60%W_peak_: 60% of peak work load achieved during IE, Voluntary pacing: Work load based on voluntary pacing, EXH: Exhaustion, P30: 30 min recovery brake, P5: 5 min brake.

Maximal and submaximal exercise performance tests were performed as described below and shown in Figure 1. All devices were calibrated according to manufacturer’s recommendations.

#### Incremental Exercise Test

The incremental exercise test was performed on an electronically braked bicycle ergometer (Ergoline Ergometrics 800, Germany). The subjects were wearing a facemask covering mouth and nose (Hans Rudolph, Kansas City, United States). Ventilation and gas exchange was measured breath by breath (Innocor, Innovision, Denmark). Additionally, subjects were monitored via a 12-lead ECG (Padsy, Medset, Germany). Subjects rested for 5 min on the bicycle. Then, the test started with a 5 min warm-up phase at a work load of 100 W (males) or 70 W (females) after which the work load was increased by 30 W every 2 min until exhaustion. During the final phase, the subjects were verbally encouraged in a standardized manner to perform to exhaustion. At peak work rate, subjects rated their perceived exertion on the 6–20 point Borg scale (Borg, 1982). The individual peak oxygen uptake (V̇O_2p__eak_) was calculated by averaging 15 s intervals during the final phase. The highest 15 s V̇O_2_ interval was considered as V̇O_2p__eak_ as proposed (Martin-Rincon et al., 2019). The same principle was applied to determine peak cardio-respiratory variables. The cardio-respiratory parameters were also related to body mass (BM). Peak work load (W_peak_) was calculated as follows: W_peak_ = W_compl_ + 30 × (t/120), W_compl_: last completed workload stage, *t*: number of seconds at final work load.

#### Submaximal Exercise

Submaximal exercise was performed after a 30 min recovery phase from the incremental test. First, baseline cardiorespiratory variables were recorded for 5 min prior to cycling for 5 min at 40% of W_peak_ achieved in the incremental test (W_40__%__peak_) followed by cycling for 5 min at 60% W_peak_ (W_60__%__peak_). At rest, data of the last 1 min prior to the start of exercise were averaged. During cycling, 2 min averages were calculated starting 1 min after the beginning of each loaded stage.

The cardiac output (CO) was measured at rest and during the final minute of each work load stage by use of the minimally invasive lithium indicator dilution technique (LiDCOplus, LiDCO cardiac sensor systems, London, United Kingdom), a method which uses lithium chloride as an indicator of cardiac output. The LiDCO plus technique has been extensively validated at rest and during exercise (Linton et al., 1993, 2000; Hadian et al., 2010; Elliott et al., 2012) and provides a comparable accuracy as the thermodilution method via Swan-Ganz catheter (Hadian et al., 2010). Briefly, a standard arterial catheter was placed into the radial artery of the subject using the Seldinger technique (Pancholy et al., 2012) under sterile precautions and local anesthesia with 1% lidocaine solution. Prior to the catheterization of the radial artery the Allen’s test was applied to guarantee redundant blood supply. To measure the CO 0.30 mmol of the non-diffusible indicator lithium chloride was injected via a peripheral venous catheter; the resulting arterial lithium chloride concentration was measured and the concentration-time curve was recorded. The cardiac index (CI) was calculated as follows: CI = CO/BSA (BSA: body surface area, du Bois formula; Du Bois and Du Bois, 1989).

#### Time Trial Performance

The submaximal exercise described above also served as warm-up for the following 20 min self-paced time trial (TT_20_), which was performed on a road bike equipped with a power meter (SRM Science power meter, Germany) and attached to an indoor trainer (Tacx, The Netherlands). Subjects were instructed to cover as much distance as possible during the 20 min. Heart rate was measured by a chest-belt heart rate monitor system (Polar Vantage NV, Oulu, Finland). Subjects’ RPE was assessed via the Borg scale after 5, 10, 15, and 20 min of cycling.

#### Venous Blood Sampling and Analysis

Venous blood samples were taken from an antecubital vein 24 h post infusion at rest prior to the start of the testing day. All blood samples were analyzed in the GLP-certified hematological laboratory of the University Hospital Zurich (Switzerland). Hematological parameters (Hb, Hct) were assessed and serum EPO concentrations were measured by use of a quantitative ELISA-assay (Human Erythropoietin Quantikine IVD ELISA Kit, R&D Systems, United States).

#### Statistical Analyses

Statistical analysis was performed using SPSS V25.0 (IBM, United States) software package, GraphPad Prism V8 (Graph Pad, United States) and R Statistics V3.5.0 software package. For statistical analysis, only data of subjects that completed exercise tests on both testing days were considered. Data were tested for normal distribution by Q-Q-plots and the Shapiro-Wilk test. The homogeneity of variances was tested with Levene’s test. A linear mixed effect model (R package: nmle) was applied to test for a main effect of treatment condition (PLA vs. EPO) on primary outcome variables considering presence of potential period (first test day vs. second test day) – or carryover (treatment sequences: PLA-EPO, EPO-PLA) effects: Y ∼ tm + pd + seq + tm ^∗^ sex + id; Y: Outcome variable. Fixed factors: tm: treatment; pd: period; seq: sequence. Random factor: id: subject identifier, factor interaction: treatment versus sex, tm ^∗^ sex.

For repeated measures a fixed time factor (t) was added to test for a time dependent treatment effect (interaction): Y ∼ tm + pd + seq + tm ^∗^ sex + tm ^∗^ t + id; Data are presented either as averages ± SD or as mentioned otherwise in the text. A two-tailed *P*-value less than 0.05 was considered significant.

## Results

### Main Characteristics of Study Subjects

In total, 29 young and healthy subjects participated in the study and main characteristics are presented in Table 1.

**TABLE 1:** Main characteristics of study patients.

**Parameter**	**Values**
N total (f/m)	29 (14/15)
Age (years)	253(21−32)
Height (cm)	1749(160−188)
Weight (kg)	659(50−81)
BMI (kg⋅m^–2^)	21.21.5(18.5−24.4)
BSA (m^2^)	1.80.2(1.5−2.0)

**Data are presented as numbers (N) or as averages ± SD (ranges). f, females; m, males; BMI, Body mass index; BSA, Body surface area.**

### Treatment and Hematological Parameters

The treatment (short infusion) was well-tolerated by the study participants and no severe adverse reactions were observed during the intervention and thereafter. The average serum erythropoietin levels 24 h post EPO treatment were almost 200-fold increased [1901 ± 659 IU⋅l^–1^ (coefficient of variation, CV: 34.7%)] in comparison to PLA [10 ± 4 IU⋅l^–1^ (CV: 46.3%)]. There was no main effect of EPO treatment on Hb [PLA: 142 ± 11 g⋅l^–1^ (CV: 7.6%) vs. EPO: 144 ± 11 g⋅l^–1^ (CV: 7.6%), *p* = 0.412) and Hct (PLA: 41.8 ± 0.0% (CV: 6.8%) vs. EPO: 42.4 ± 0.0% (CV: 6.5%), *p* = 0.410] at this time point. Of note, a period effect was present for both hematological variables (Hb, *p* = 0.010, Hct, p = 0.018) with higher values observed in the first test period in comparison to the second period. However, the frequency of treatment condition at each period was balanced and no treatment effect was found for Hb (first test day, *p* = 0.954, second test day, *p* = 0.598) and Hct (first test day, *p* = 0.743, second test day, *p* = 0.678) when analyzing each period separately. Furthermore, no carryover effect was found for these parameters (Hb, *p* = 0.595, Hct, *p* = 0.882).

### Peak Exercise Capacity

**FIGURE 2:**
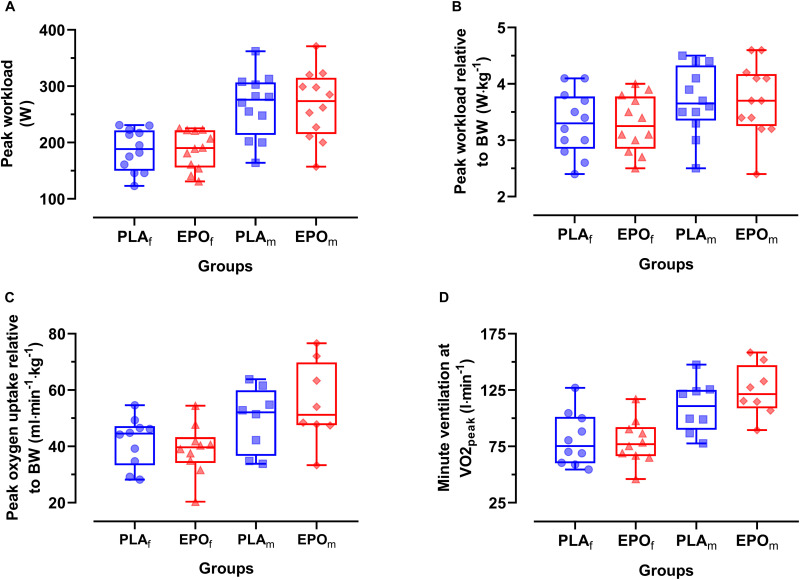
Primary outcome parameters of the incremental cycling test divided by sex (*N* = 24). No main effects of EPO treatment were observed for any of the presented outcome parameters. However, an interaction effect between treatment and sex was found for V̇E_peak_ leading to relatively (vs. PLA) higher values in EPO-treated males in comparison to females. **(A)** Peak workload (W_peak_), **(B)** Peak work load relative to body mass (BM), **(C)** Peak oxygen uptake relative to BM, **(D)** Minute ventilation at V̇O_2p__eak_ (V̇E_peak_). Groups: EPO_f_ (red triangles): High dose (60.000 IU per person) EPO treated females, PLA_f_ (blue circles): Placebo-treated females, PLA_m_ (blue squares): PLA-treated male subjects, EPO_m_ (red rhomboid squares): EPO-treated male subjects.

The main outcome parameters of the maximal incremental cycling test are shown in Figure 2. There was no main effect of EPO treatment on peak work load (W_peak_) (PLA: 226 ± 61 W vs. EPO: 227 ± 63 W, *p* = 0.952), W_peak_ relative to body mass (PLA: 3.5 ± 0.6 W⋅kg^–1^ vs. EPO: 3.5 ± 0.6 W⋅kg^–1^, *p* = 0.892), peak oxygen uptake relative to body mass (PLA: 45.1 ± 10.4 ml⋅min^–1^⋅kg^–1^ vs. EPO: 46.1 ± 14.2 ml⋅min^–1^⋅kg^–1^, *p* = 0.344) and minute ventilation at V̇O_2p__eak_ (PLA: 94.2 ± 27.1 l⋅min^–1^ vs. EPO: 99.4 ± 31.0 l⋅min^–1^, *p* = 0.679). No main effect of EPO treatment was observed in the respiratory exchange ratio at W_peak_ (PLA: 1.11 ± 0.08 vs. EPO: 1.12 ± 0.07, *p* = 0.685) and in the rating of perceived exertion at W_peak_ (PLA: 18.6 ± 1.1 vs. EPO: 18.9 ± 1.3, p = 0.096). However, there was an interaction effect between the factors treatment and sex for V̇O_2p__eak_ (*p* = 0.048) and minute ventilation at V̇O_2p__eak_ (*p* = 0.044) toward higher values in EPO-treated males.

### Submaximal Exercise Testing

Data during submaximal exercise prior to the time trial are summarized in Table 2. There was no main effect of EPO treatment on primary outcome parameters (V̇E, V̇O_2_, V̇O_2_/BM, HR, MAP, CO, CO/BM, CI) at baseline nor at 40 or 60% of W_peak_. Additionally, no interaction was observed between the factors treatment and sex.

**TABLE 2:** Main outcome variables during submaximal exercise.

	**Rest**	**TME**	**W_40__%__peak_**	**TME**	**W_60__%__peak_**	**TME**
**Variable**	**PLA vs. EPO**	***P*-value**	**PLA vs. EPO**	***P*-value**	**PLA vs. EPO**	***P*-value**
V̇O_2_	0.40.1	0.214	1.60.4	0.447	2.10.6	0.756
(l⋅min^–1^)	0.60.3		1.80.6		2.30.8	
V̇O_2_/BM	6.61.5	0.092	25.23.7	0.905	33.15.3	0.905
(ml⋅min^–1^⋅kg^–1^)	9.15.4		27.98.7		37.113.2	
V̇E	15.66.4	0.055	39.56.9	0.439	57.69.8	0.421
(l⋅min^–1^)	19.97.4		42.113.4		61.121.6	
HR	9613	0.676	13015	0.098	15611	0.631
(beats⋅min^–1^)	9813		14015		15615	
MAP	854	0.311	9310	0.522	1016	0.756
(mmHg)	867		957		1018	
CO	5.61.7	0.961	11.33.4	0.875	14.85.8	0.207
(l⋅min^–1^)	5.41.1		11.03.6		13.24.2	
CO/BM	8719	0.900	17842	0.658	23172	0.134
(ml⋅min^–1^⋅kg^–1^)	8511		16937		20444	
CI	3.10.8	0.946	6.41.6	0.750	8.32.8	0.148
(l⋅min^–1^⋅m^–2^)	3.10.4		6.11.5		7.41.8	

**Data are presented as means ± SD, V̇O_2_, Oxygen uptake; V̇O_2_/BM, Oxygen uptake relative to body mass; V̇E, Minute ventilation; HR, Heart rate; MAP, Mean arterial pressure; CO, Cardiac output; CO/BM, Cardiac output relative to body mass; CI, Cardiac index; TME, Treatment effect (N = 19).**

### Time Trial Performance (TT_20_)

**FIGURE 3:**
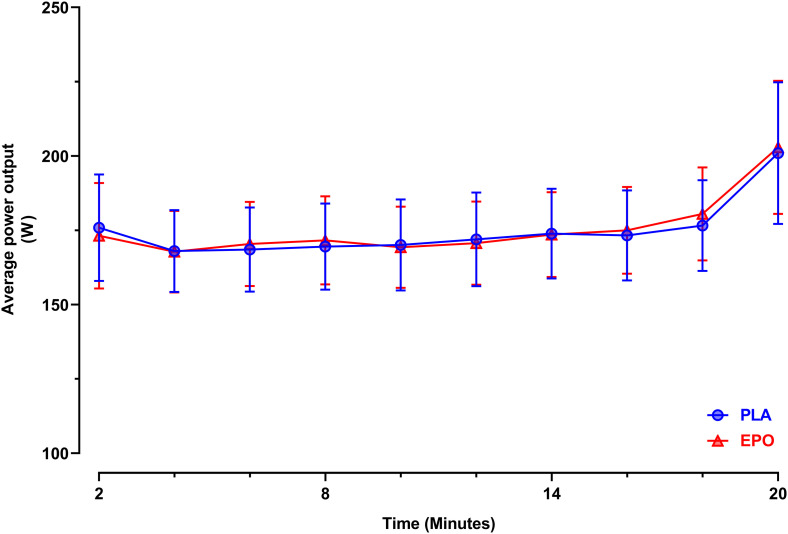
Average power output during the 20 min time trial (*N* = 25). Data are presented as 2 min averages ± 95% confidence intervals (95%CI). TT_20_: 20 min time trial, EPO (red triangles): High dose (60.000 IU per person) EPO-treated subjects, PLA (blue circles): Placebo-treated subjects.

**FIGURE 4:**
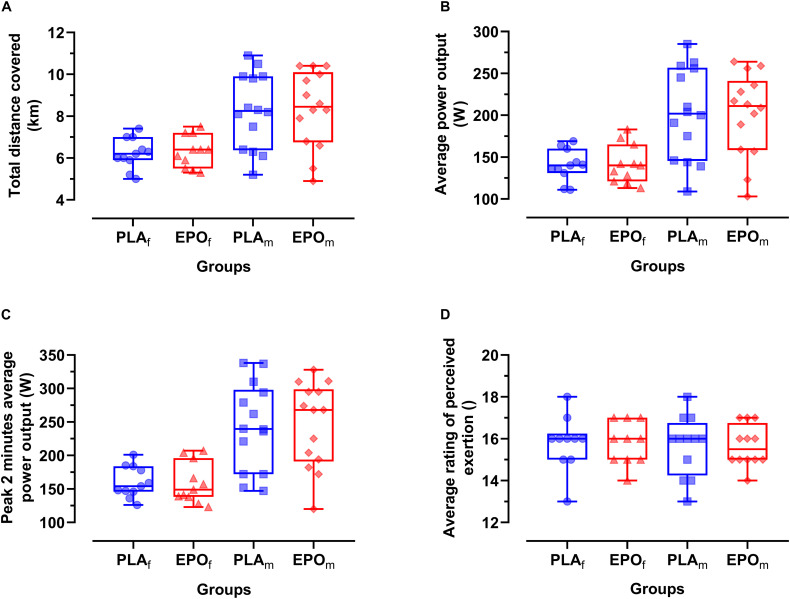
Primary outcome parameters of TT_20_ divided by sex (*N* = 25). No main effects of EPO treatment and no interaction effects between treatment and sex were observed for any of the presented outcome parameters. **(A)** Total time trial distance (D), **(B)** average power output (W_avg_), **(C)** Peak 2 min average power output (W_avg__2p__eak_), **(D)** average rating of perceived exertion (RPE_avg_). Groups: EPO_f_ (red triangles): High dose (60.000 IU per person) EPO treated females, PLA_f_ (blue circles): Placebo-treated females, PLA_m_ (blue squares): PLA-treated males, EPO_m_ (red rhomboid squares): EPO-treated males.

Figure 3 depicts the time-course of the average power in the course of the trial. Primary outcome parameters of the TT_20_ are summarized in Figure 4. There was no main effect of EPO treatment on total distance covered (PLA: 7.4 ± 1.7 km vs. EPO: 7.4 ± 1.8 km, *p* = 0.527), average power output (PLA: 175 ± 52 W vs. EPO: 175 ± 50 W, *p* = 0.763) and average rating of perceived exertion (PLA: 15 ± 1 vs. EPO: 15 ± 1, *p* = 1.000). Overall, there was no interaction effect between the factors treatment and sex for any of the aforementioned outcome parameters.

### Subjective Rating of Sleep Quality, Mood/Motivation *Prior to Performance Testing*

No main effect of EPO treatment was observed in sleep quality after treatment (PLA: 75 ± 19% vs. EPO: 73 ± 20%, *p* = 0.749), mood rating (PLA: 81 ± 12% vs. EPO: 80 ± 14%, *p* = 0.801) and motivation (PLA: 79 ± 13% vs. EPO: 79 ± 15%, *p* = 0.992). Also, no main effect of EPO treatment was detected for PAS (PLA: 37.8 ± 6.2 vs. EPO: 38.8 ± 7.3, *p* = 0.670) or NAS (PLA: 11.9 ± 2.5 vs. EPO: 12.0 ± 2.7, *p* = 0.475). Furthermore, there was no interaction effect between the factors treatment and sex for any of the aforementioned subjective ratings.

## Discussion

In the present study, we found a single very high dose of EPO (60.000 IU) to neither affect peak exercise capacity nor endurance exercise performance. In addition, there was no sex-related difference in these outcomes. Sleep quality, mood and motivation 24 h after treatment and prior to performance testing remained unaffected by the treatment.

### High Dose of EPO Treatment on Peak Aerobic Exercise Performance

Maximal oxygen uptake (V̇O_2m__ax_) assessed during incremental cycling is a key determinant of aerobic exercise capacity and reflects the ability to provide oxygen to the exercising skeletal muscles (Bassett and Howley, 2000) while its magnitude is predicted by cardio-respiratory and circulatory parameters such as cardiac output and red blood cell volume (Lundby et al., 2017). V̇O_2m__ax_ is also a strong predictor of all-cause mortality in men and women (Kodama et al., 2009). In the present study, we referred to the term V̇O_2p__eak_ instead of V̇O_2m__ax_ as the V̇O_2_-work rate plateau, required to assess “true” V̇O_2m__ax_ values is frequently absent in an incremental exercise test setting (Poole and Jones, 2017). Although serum EPO levels were increased 200-fold during testing 24 h after administration of a single dose of EPO, peak aerobic exercise capacity did not improve in the present study as shown in unaffected W_peak_ and V̇O_2p__eak_ in the incremental cycling test. This finding contrasts with a study performed in mice (Schuler et al., 2012) where V̇O_2m__ax_ during an incremental exercise test was increased in wild-type mice after a single injection of high dose EPO leading to increased brain EPO levels without affecting hematological parameters. While, in humans, prolonged EPO treatment over several weeks is well known to improve V̇O_2p__eak_ (Lundby and Olsen, 2011; Sgro et al., 2018) the aforementioned animal study was the first to demonstrate a potential acute (within 4–6 h) ergogenic effect of EPO, independent of hematological changes (Schuler et al., 2012). In the absence of alterations in measured cardio-respiratory (e.g., cardiac output) and hematological parameters (e.g., total hemoglobin mass) that could have explained the observed EPO effect, it was hypothesized that EPO may exert its acute ergogenic effect by a central mechanism within the brain, possibly supported by a tendency toward a change in RER at peak exercise (Schuler et al., 2012). Furthermore, the same exercise test setting was used to test transgenic mice (Tg21), which constitutively overexpress EPO in a hypoxia-independent manner solely in the brain while having normal hematological values. In support of the expectations, the Tg21 mice also reached higher V̇O_2m__ax_ levels during the incremental exercise test as compared to their wild-type littermates (Schuler et al., 2012). In humans, motivational processes and changes in mood state were previously linked to performance (Lane and Terry, 1999; Lane et al., 2017; Taylor et al., 2020). The study of Miskowiak et al. (Miskowiak et al., 2008) provided a functional basis for a central performance enhancing effect of EPO by showing that a single high dose of EPO (40.000 IU) treatment improved self-reported mood in healthy subjects for three consecutive days. The self-reported mood state was assessed by use of the positive and negative association scale, PANAS (Watson et al., 1988). We also assessed the self-reported mood by use of PANAS, 24 h after the treatment and prior to performance testing. However, we did not observe a main treatment effect of EPO on positive and negative associations of PANAS. Furthermore, subjective rating of mood state, motivation level and sleep quality were not affected by the treatment either. Following the hypothesis of a central EPO effect, the absence of changes in mood state and motivation observed in our study setting may explain why EPO did not increase peak aerobic exercise performance. In line with the unchanged performance is the lack of change in peak respiratory exchange ratio and rate of perceived exertion. However, it remains generally questionable whether the results obtained from incremental exercise tests in rodents are directly translatable to those in humans. One important contrasting aspect is the obvious lack of feedback options to evaluate the reasons for discontinuation of incremental exercise in rodents. Another aspect, which was also mentioned by the authors of the mouse study (Schuler et al., 2012) is the limited time resolution of measuring metabolic parameters (e.g., V̇O_2_ and V̇CO_2_) during incremental exercise testing in rodents in contrast to breath by breath resolution in humans.

The present results are in line with findings of another human study using short-term high dose EPO (30.000 IU⋅day^–1^ for 3 consecutive days) that also did not find an increased performance in an incremental exercise test to exhaustion (Rasmussen et al., 2010). Furthermore, those authors reported that EPO did not alter central fatigue expression and concluded that EPO does not affect central motor drive. Of note, we observed an interaction effect of treatment and sex for V̇O_2p__eak_ with a tendency for higher values in EPO-treated males and lower in females. Here, it needs to be mentioned that previous studies, which evaluated the potential impact of short-term high dose EPO treatment on exercise performance in rodents (Schuler et al., 2012) and humans (Rasmussen et al., 2010) only included males. However, to which extent the observed effect in our study is a coincidental finding or a true sex-dependent metabolic difference, certainly needs more specific investigations.

### High Dose of EPO Treatment on Cardiorespiratory Measures During Submaximal Exercise

To elucidate the effect of a single high dose of EPO on cardiorespiratory parameters of exercise performance at the submaximal level, we assessed these at rest, and during cycling at 40 and 60% of W_peak_. As for maximal exercise variables, no main treatment effect of EPO was present in any of the parameters. Also, no interaction effect between the factors treatment and sex was found. However, there was a tendency toward higher values in V̇O_2_/BM and V̇E in EPO-treated subjects at resting conditions, which may indicate an altered metabolic state in these subjects. Indeed, a recent study reported that a single high dose of EPO (400 IU⋅kg^–1^) can acutely stimulate resting energy expenditure in healthy subjects and this effect was accompanied by a tendency toward increased fat oxidation while glucose and protein metabolism remained unchanged (Christensen et al., 2012). However, the present study was not designed to address the potential effects of high dose EPO treatment on energy metabolism at resting conditions and during exercise. Nevertheless, numerous evidence from preclinical and clinical studies suggest that EPO alters energy metabolism and energy homeostasis at different regulatory levels (summarized in Wang et al., 2014). Interestingly, the observed EPO effect on V̇O_2_/BM and V̇E at rest appeared to vanish during the two different intensity levels of submaximal exercise. Furthermore, an increased variability as reflected by an increased standard deviation was observed for both parameters at both intensities in EPO-treated subjects, which may indicate an increased response heterogeneity of subjects to EPO treatment and may have masked a potential EPO effect. The human trial performed by Rasmussen et al. (Rasmussen et al., 2010) revealed that short-term high dose EPO treatment (1 × 30.000 IU⋅day^–1^ for 3 consecutive days) increased exercise V̇E in healthy young man during low intensity exercise testing but as stated previously did not increase exercise capacity. Another potential reason to why we did not observe this EPO effect on V̇E during low intensity (40% of W_peak_) stage of submaximal exercise could be that a single high dose of EPO treatment was insufficient to induce a comparable effect magnitude on exercise ventilation. Thus, it needs to be clarified at which submaximal exercise intensities and after which administration regimes of high dose EPO treatment these effects on exercise ventilation occur.

### High Dose of EPO Treatment on Time Trial Performance

Also for a competition-like cycling test that was expected to be most prone to central changes, e.g., changes in motivation, we did not find a main treatment effect of EPO and no interaction effect between treatment and sex was found. The pacing strategy was similar as well, as seen in the time-course of the power achieved. In case of a positive central effect, we had hypothesized that the start or end spurt would be more pronounced after EPO treatment. Average rating of perceived exertion also remained unaffected by the treatment. These results, again contrast findings of Schuler et al. (2012) who reported an increased time to exhaustion as an indicator of improved endurance exercise capacity in mice treated with a single high dose of EPO during a constant-load test performed at 80% of maximal work load to exhaustion but as discussed before, no cardio-respiratory changes could explain these findings (Schuler et al., 2012).

### Limitations of the Study

The current study has certain limitations. First, cerebral EPO levels, i.e., levels in the cerebrospinal fluid were not quantified due to the invasive nature of the procedure and since comparable high doses of EPO were already shown to yield increased erythropoietin levels in the CSF of humans (Rasmussen et al., 2010). Second, in females, exercise testing might have taken place in different phases of the menstrual cycle of for logistical reasons which might have slightly interfered with measured cardiorespiratory parameters, especially during submaximal exercise testing (Oosthuyse and Bosch, 2010). However, the vast majority (>90%) of participating females were using oral contraception throughout the testing period, which was recently shown not to affect peak oxygen uptake and exercise performance remained unaffected (Quinn et al., 2018). Furthermore, the applied statistical model was designed to test for the presence of potential period effects and also for potential interaction effects between the factors treatment and sex on primary outcome variables. Third, we cannot exclude sub-effect threshold treatment due to the lack of available preclinical dose-response data about short-term high dose EPO treatment and exercise performance. Indeed, the reported EPO dose administered in mice (∼77.000 IU⋅kg^–1^ of body mass) (Schuler et al., 2012) was “extremely” high and exceeded the EPO dose administered in humans (60.000 IU⋅per person, ∼800 IU⋅kg^–1^ body mass, referred to a 75 kg person) nearly by a factor of 100. This topic needs to be further investigated and most importantly it needs to be taken into account that the risk of adverse events may also change in dependence of the EPO dose administered. Here, a bedside to bench approach may provide more insight.

## Conclusion

A single high dose of EPO treatment did not improve aerobic exercise capacity in healthy humans but modulated peak oxygen uptake and exercise ventilation in a sex-dependent manner.

## Data Availability Statement

The datasets generated for this study are available on request to the corresponding author.

## Ethics Statement

The studies involving human participants were reviewed and approved by the Cantonal Ethical Commission of Zurich (KEK-ZH-NR: 2011-0170). The patients/participants provided their written informed consent to participate in this study.

## Author Contributions

TH and VD designed the study protocol. TH, VD, JA, MA-S, MT, and MH performed the experiments. TH performed the statistical analyzes, wrote, and edited the manuscript. VD, MT, MM, MH, CS, and MG revised the manuscript. All authors contributed to the article and approved the submitted version.

## Conflict of Interest

The authors declare that the research was conducted in the absence of any commercial or financial relationships that could be construed as a potential conflict of interest.
